# Investigation of the precision of the Visia^®^ complexion analysis camera system in the assessment of skin surface features

**DOI:** 10.3205/iprs000169

**Published:** 2022-11-29

**Authors:** Helga Henseler

**Affiliations:** 1 Klinik am Rhein, Klinik für Plastische und Ästhetische Chirurgie, Düsseldorf, Germany

**Keywords:** Visia® camera, precision, assessment, skin surface, imaging

## Abstract

**Background:**

The aim of this study was to independently investigate the precision of the high resolution Visia^®^ camera, from Canfield Scientific, to capture several skin surface features.

**Method:**

Facial images of eight volunteers were taken with closed eyes and a relaxed face. The capture was conducted in a resting position within a positioning rig. Frontal view images were taken. In the first capture session, the images were captured three times in a row with the head steadily resting in the capture rig. Each volunteer then left the capture rig and returned to it one week later repositioning the face, and the capture was repeated three times. On the basis of this study, it was additionally investigated which number of study participants would be required in order to make a claim as to the reproducibility of the captures. As a possible approach to making this determination, a power analysis was considered. In order to conduct this analysis, it was necessary to determine which differences between individual image captures would be clinically acceptable. To answer this question, a subjective assessment of the repeated image captures for all study participants and for all skin surface features was conducted in order to identify any differences that were visible with the human eye.

**Results:**

Differences in skin criteria of the eight volunteers in terms of means and standard deviations were collected for weeks one and two. For the criteria skin texture, UV spots, brown spots and porphyrins, these differences were less than 2% and for pores and red areas they were between 2% and 4%. The results for spots and wrinkles were around 6%. Looking at the differences between the data from week one and two as well as the standard deviations, these differences turned out to be relatively small. This finding also pointed to a quite good precision of the measurement technique. The subjective assessment of the images of the eight participants on each of the eight skin criteria revealed that no differences were detectable in the recaptured versions of the images of the participants’ faces in their native digital forms solely with the human eye. There was an exception for only one participant, in whom a distinction between two image captures appeared to be subjectively visible with the human eye, but only for the criterion of red areas. As the subjective assessment revealed that no clinically relevant differences could be identified, a power analysis involving a test for significant differences between the recaptured images was discarded. As a consequence, the number of participants recruited for the study on the reproducibility of the system presented herein was deemed to be sufficient.

**Conclusion:**

The precision of the Visia^®^ camera system was found to be satisfactory in this study. The Visia^®^ camera helped to visualise skin features beyond what is visible to the human eye. Thus, the Visia^®^ camera system provides new objective information on skin surface characteristics beyond what can be acquired through purely subjective assessments.

## Zusammenfassung

**Einleitung:**

Das Ziel der Studie war eine unabhängige Untersuchung der Genauigkeit der hochauflösenden Visia^®^ Kamera der Firma Canfield Scientific für die Bildaufnahmen von mehreren Hautoberflächeneigenschaften.

**Methode:**

Gesichtsaufnahmen von acht Freiwilligen wurden mit geschlossenen Augen und einem entspannten Gesicht erhoben. Die Bildaufnahmen wurden in einer Ruheposition innerhalb eines Positionierungsrahmens durchgeführt. Es wurden Frontalansichten aufgenommen. Bei der ersten Bildaufnahmesitzung wurden die Aufnahmen dreimal hintereinander aufgenommen, wobei der Kopf im Positionierungsrahmen ruhte. Jeder Freiwillige verließ dann das Aufnahmegestell und kehrte eine Woche später wieder zu diesem zurück und repositionierte sein Gesicht, und die Bildaufnahmen wurden dreimal wiederholt. Weiterhin wurde basierend auf diesen Studiendaten untersucht, wie groß die notwendige Anzahl an Probanden sein sollte, um eine Aussage zur Reproduzierbarkeit der Bildaufnahmen machen zu können. Zu diesem Zweck wurde die Durchführung einer Power-Analyse in Erwägung gezogen. Die Frage lautete: Welcher Unterschied wäre klinisch akzeptierbar? Um diese Frage zu beantworten, erfolgte eine subjektive Untersuchung der wiederholten Bildaufnahmen aller Studienprobanden im Hinblick auf alle Hauteigenschaften, um festzustellen, welche Unterschiede mit dem bloßen menschlichen Auge sichtbar wären.

**Ergebnisse:**

Die Unterschiede der Hauteigenschaften der acht Freiwilligen im Hinblick auf Durchschnittswerte und Standardabweichungen wurden für Woche eins und zwei erhoben. Für die Kriterien Hauttextur, UV Flecken, braune Flecken und Porphyrine betrugen diese Unterschiede weniger als 2%, für Poren und rote Areale zwischen 2% und 4%. Die Ergebnisse für Flecken und Falten lagen bei 6%. Bei der Betrachtung der Unterschiede zwischen den Daten aus Woche eins und zwei sowie der Unterschiede der Standardabweichungen ergab sich, dass diese Unterschiede relativ klein waren. Dieses Ergebnis weist auch auf eine recht gute Präzision der Messtechnik hin. Die subjektive Untersuchung jeder einzelnen der acht Hauteigenschaften der acht Studienprobanden ergab, dass keine Unterschiede mit dem bloßen menschlichen Auge auf den nativen, digitalen, wiederholten Bildaufnahmen der Gesichter erkennbar waren. Nur bei einem einzigen Teilnehmer schien mit dem menschlichen Auge ein Unterschied zwischen zwei Bildaufnahmen subjektiv sichtbar zu sein, einzig für das Kriterium der roten Areale. Da die subjektive Beurteilung ergab, dass keine klinisch relevanten Unterschiede gefunden werden konnten, wurde eine Power-Analyse, die einen Test der Signifikanzen von Unterschieden zwischen den wiederholten Bildaufnahmen beinhaltet, verworfen. Folglich wurde die Anzahl der Probanden für die hier präsentierte Studie zur Reproduzierbarkeit der Bildaufnahmen des Systems als ausreichend ermittelt.

**Schlussfolgerung:**

Die Genauigkeit des Visia^®^ Kamerasystems erwies sich in dieser Studie als zufriedenstellend. Die Visia^®^ Kamera half Hauteigenschaften jenseits dessen, was mit dem menschlichen Auge sichtbar ist, zu visualisieren. Das Visia^®^ Kamerasystem gibt neue objektive Informationen zu Eigenschaften der Hautoberfläche jenseits rein subjektiver Untersuchungen.

## Introduction

Imaging in plastic surgery has traditionally been considered important for depicting and documenting conditions of the body or facial surface conditions [1], [2]. Photographic documentation served to discuss patient wishes and display conditions before and after surgery, for quality control needs as well as teaching and training purposes [3]. Plastic surgeons tended to keep multiple photographic files, which was considered essential to the practice of plastic surgery [2], and important medical-legal purposes were cited [1].

In order to enable comparisons pre- and postoperative photographs should be standardised [1], [3]. Guidelines were published on the correct procedure for facial photography [4]. To date, digital photography has most completely replaced previous film photography [5]. Additionally, data protection requirements must be strictly observed [4], [6], [7].

The development of high-resolution camera systems has been important for improving image quality [8], [9], [10] and the focus and the depth of the field in medical digital imaging have been subjects of research [11]. In the meantime, several suppliers have equipped their cameras for plastic surgery use, in some cases with complex analysis software. In most cases, however, these camera systems were complex and not validated [12]. The reasons for this lack of validation studies and according publications can be found in the complexity of the required investigations.

One of these modern high-resolution digital camera systems, called the Visia^®^, from Canfield Scientific, Inc., New Jersey, USA (https://www.canfieldsci.com), was used for facial capture and analysis. The Visia^®^ is a commercialised clinical imaging device. While the Visia^®^ camera system has been used in a few studies for clinical evaluations, there have been few independent studies and publications about the imaging system itself. 

It is known from previous research that when investigating the validity of an imaging system, accuracy and reproducibility as well as errors should be examined and that all the different aspects contribute to the overall picture [8], [9], [10].

The present study focuses on the independent investigation of the reproducibility, which has not yet been presented. 

## Aim

The investigation aimed to independently assess the precision of the Visia^®^ camera system by assessment of the reproducibility with its given aspects of facial skin surface characteristics. 

## Method

**Figure 1:**
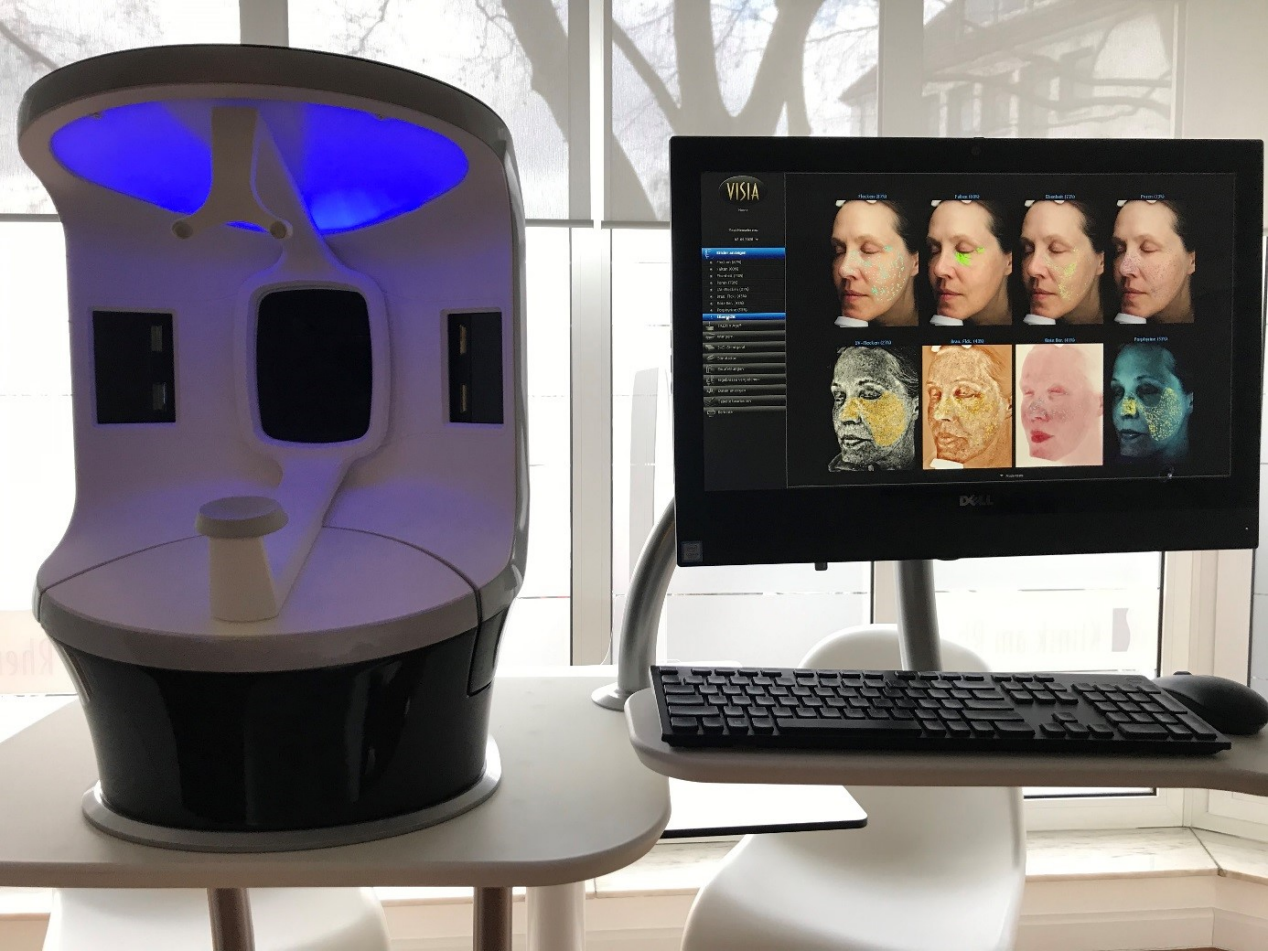
The Visia^®^ complexion analysis camera system

The Visia^®^ Complexion Analysis Camera is a camera system for displaying various skin surface characteristics (Figure 1 (Fig. 1)). In the majority of cases, these surface characteristics are recorded in a two-dimensional way. However, the system also offers the possibility to display certain areas of interest in a three-dimensional way. In general, eight skin characteristics are displayed:

1. spots on the skin surface that are brown or red marks,
2. wrinkles in the skin such as fine lines or deeper folds,
3. skin texture recorded as raised or depressed surface areas,
4. pores visible as circular openings in the skin,
5. UV spots indicating sun damage due to absorption of UV light by epidermal melanin,
6. brown spots that display any brown mark on the skin either as a natural brown mark or a sun damage,
7. red areas that can display blood vessels and haemoglobin and can be related to a variety of skin conditions and
8. porphyrins, which are the bacterial excretions that can be found in the pores produced by propionibacterium acnes.

For the display of the various skin surface features different lighting is required such as standardised lighting (features 1–4), cross-polarised lighting (features 5–7) and fluorescence under UV light (feature 8). For analysis, the software measures data which are displayed in percentile scores. These represent the skin feature in a masked area in comparison to a data-base of skin features of a group of people of the same age and skin type. The higher the score, the fewer problems the subject has, with 50% representing the average of the reference group.

Eight volunteers were recruited from staff of a plastic surgical clinic. Recruitment was conducted by verbal approach by the lead researcher within the clinic and information to the purpose and conduct of the study was provided. Informed consent was obtained. Inclusion criteria were that consent was given, volunteers understood the purpose of the capture and the question that should be answered and a clean skin surface without make-up. Exclusion criteria were volunteers that did not consent, hypersensitivity to flashes even when eyes were closed during the capture, which always was the case, epilepsy, claustrophobia or make-up on the skin surface. The demographic data of the volunteers for capture are given (Table 1 (Tab. 1)). 

Frontal facial images of eight volunteers were taken with clean skin, closed eyes and a relaxed face following a standardised capture protocol. The capture was conducted in a resting position with a positioning rig to stabilise the facial posture. In the first capture session, the capture was conducted three times in a row with the head steadily resting in the capture rig. Each volunteer then left the capture rig and returned to it one week later with repositioning of the face and the capture was repeated three times as before. By automated software calculation the images were realigned with the previous ones and an analysis of the images was conducted. 

Data were collected and means and standard deviations from the three repeated measurements of the skin features were obtained. Standard deviations were used as a measure of the precision of the recording of each of the quoted skin features in week one and two. The calculation was conducted by a spreadsheet calculation program (LibreOffice Calc). The images were created with the packet “ggplot” from the program R (R Core Team (2016) (https://www.R-project.org)).

On the basis of this study, a power analysis was considered as a potential means of establishing a statistical judgment of the required number of study participants. A power analysis helps to determine the optimal sample size to ensure sufficient probability of detecting the effect of interest, via the relationships between four relevant variables: the effect size, sample size, threshold for significance, and required level of statistical power itself. In order to make the necessary calculation, it was necessary to determine what kind of differences would be clinically acceptable. 

In order to answer this question, an additional study was conducted, in which subjective assessments of the repeated captures of all study participants were carried out to determine the differences that were visible with the human eye. The native digital images of the participants’ faces captured by the Visia^®^ camera as part of the repeated captures were reviewed by the researcher in view of all eight skin criteria to determine whether differences between the images could be detected. The native images captured by the Visia^®^ camera were taken to be the initially-captured digital facial images, without the colour-coded visualisations of the various aspects of the skin that could be obtained by submitting multiple flash images captured by the Visia^®^ camera to analysis in the accompanying software package. The objective of the power analysis was to compute the required sample size, given an alpha significance threshold of 0.05 (as most commonly employed in statistics), to achieve a statistical power level (i.e., the probability of detecting an effect, given that one in fact exists) of at least 80%, which is in line with general expectations. To carry out this computation, an additional variable is needed: specifically, the statistical effect size, which is a measure of the strength of a relationship between two variables on a numerical scale. However, to determine the relevant effect size, it would be necessary to establish a definition of a clinically relevant difference.

## Results

Table 2 (Tab. 2) contains the statistical parameters of the percentile data of all eight examined persons regarding each of the eight skin features. Given are the means and standard deviations, both derived from all eight images for evaluation, each for week one and two. 

The differences of the skin criteria for the eight volunteers in view to means and standard deviations were successfully obtained for week one and two. Clearly visible became the differences between the criteria regarding the standard deviations. For the criteria skin texture, UV spots, brown spots and porphyrins, these differences were less than 2%. For pores and red areas, the standard deviations were between 2% and 4%. The results for spots and wrinkles were about 6%. 

**Figure 2:**
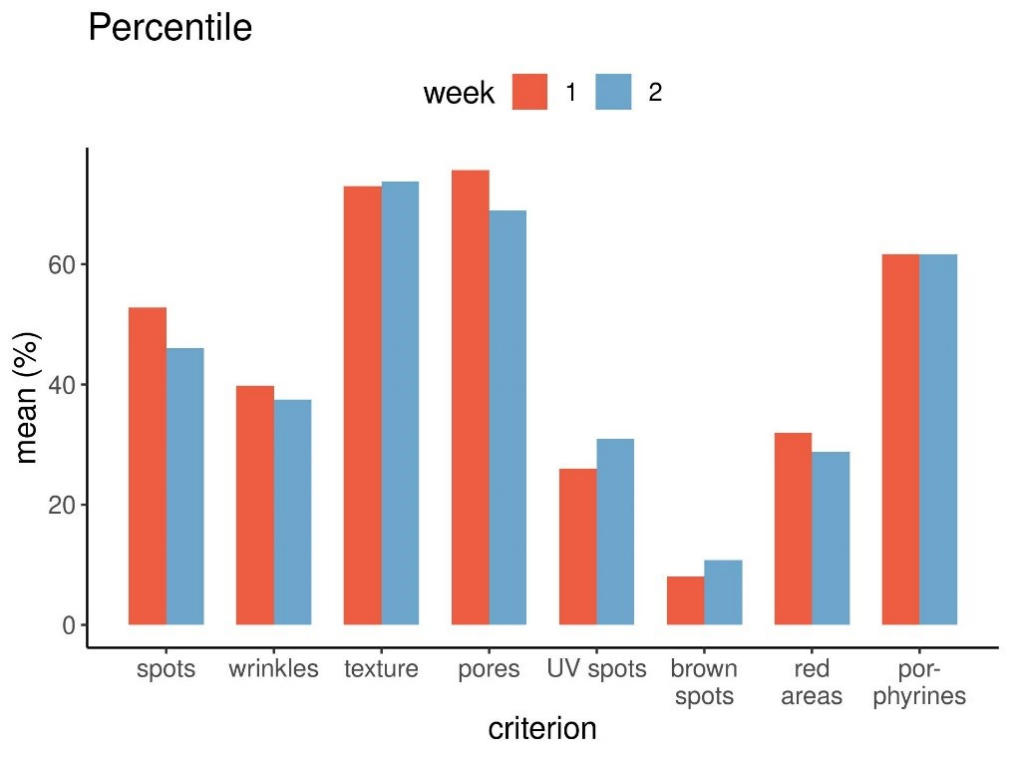
Percentile data as means for all eight persons for each of the eight criteria, week 1 and 2

**Figure 3:**
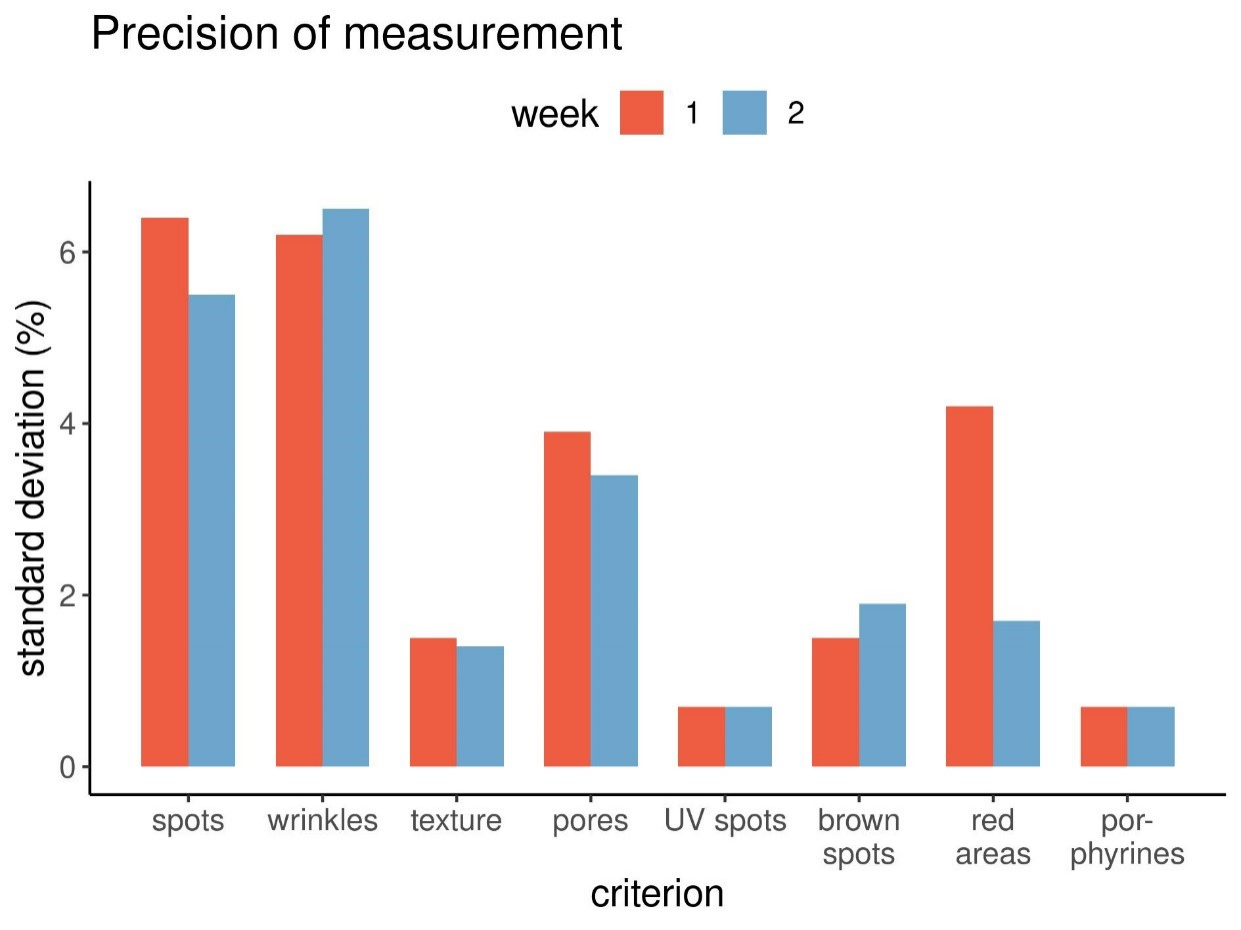
Precision of measurements as standard deviations for each criterion, week 1 and 2

Looking at the differences between the data given as percentiles between week one and two (Figure 2 (Fig. 2)) as well as the standard deviations (Figure 3 (Fig. 3)), these differences turned out to be relatively small. This finding also pointed to a quite good precision of the measurement technique.

One exception was the standard deviation of the red areas, which was 4.2% in the first week and 1.7% in the second week. Reason for this was the fact that in week one the percentile data of two volunteers varied much more between capture one, two and three than in week two. This could be due to random effects. 

In the case of each of the eight skin criteria across all eight study participants, no differences between repeated captures in the native digital facial images were found to be detectable with the human eye. In only a single participant, and only in relation to the criterion of red areas, a very slight difference was visible with the human eye: specifically, the redness appeared to be more pronounced on the nose in one capture in comparison to a previous one.

Therefore, it was determined that it was not necessary to conduct a power analysis if there was no clinical effect, and it would not make sense to conduct a test for significant differences if there were no such differences. As a consequence, the number of participants was found to be satisfactory for the investigation of the reproducibility of the system presented herein.

## Discussion

The study presented here focused on the investigation of the reproducibility of the Visia^®^ camera system and thus the precision of the system. The question could arise as to why the accuracy of the system was not also investigated. The answer lies in the different point of interest in the study presented here and the complexity of the studies required for investigating the accuracy of the multiple skin aspects. Originally, the complexion analysis software used in the Visia^®^ camera was developed by the American company Procter & Gamble in the late 1990s. The software has been used as a sales tool by P & G since 1998 and was not intended for clinical trials (https://www.canfieldsci.com/). According to the company, the analysis algorithms are supported by research work, but after closer examination there seems to be a lack of publications. 

In reviewing the literature only a few studies could be identified in which the Visia^®^ camera was mentioned to be used to investigate a dermatological problem, which as such was the focus of the study. In general, only one single skin feature of interest was examined and the images of the Visia^®^ camera were used for comparison purposes. 

In a recent study, funded by Procter and Gamble, the size of skin pores was examined in detail by 2D skin surface imaging by the Visia^®^ camera. The results were compared with subjective visual classification, which showed a high correlation [13]. The authors claimed that they had validated the novel imaging method for skin pore analysis, but not in terms of pore depth or volume. The limitations, however, were the small area of interest that was investigated, the lack of a gold standard for comparisons and the lack of assessment of deviations and errors [10], [12]. The Visia^®^ capture system, as such, was only briefly presented in this publication, which focused on pore analysis, and was not examined further. 

The Visia^®^ camera was also clinically utilised in the assessment of acne scarring after poly-L-lactic acid injection [14]. In this study, the Visia^®^ camera was cited by the authors to be the most sensitive camera system providing the most detailed imaging of skin topography. Comparisons were conducted with images from two less precise camera systems and with subjective assessments using scoring systems. An improvement in acne scarring was found, as determined by physicians, blinded evaluators and subjective assessment. The Visia^®^ system as such was not the focus of the study. 

Furthermore, a clinical variation of treatments of acne scarring by different acids was assessed in two study groups, and one of several methods of evaluation was carried out using the Visia^®^ camera system [15]. Here too, the treatment of the skin condition was the focus of interest and not the Visia^®^ camera system as a visualization or measurement instrument. 

In contrast, the study presented here investigated the precision of the Visia^®^ camera system itself. The investigation of the reproducibility is new and has not been published before. The data by software calculation are presented as percentiles by the Visia^®^ camera system. Percentiles provide a tool for the evaluation of a person’s complexion analysis results through comparison of the subject’s absolute scores to those of a group of people from a data base with similar characteristics. This method provides a basic assessment of the general complexion of the person. The higher the percentile score is, the less problems the patient has. 

The statistical method chosen here for calculating the data by determining the standard deviation of repeated tests was suitable for making a statement about the precision of the analysis. In contrast, the variation coefficient, which is the quotient of the standard deviation and the mean value, was not chosen to be calculated. This is because it is only used when the spread of data is compared in cases when the spread increases with the size of the measurements. In this study, this has not been the case. Therefore, the standard deviation was the correct measure to investigate the precision of the data. 

The results showed that there were some differences between the capture sessions. Even when the face was stabilised in the capture rig and not moving, there was variation of the results for the eight given skin surface features between repeated measurements. Variation was also obtained when retaking the pose for repeated capture. Furthermore, the results varied between the eight surface features. This level of variation could be measured and quantified for each single feature as well as between the features and the volunteers. The Visia^®^ camera was used to visualise and quantify skin features that otherwise would have remained undetectable. 

The variation in measurements presented here is consistent with previous studies of other objective capture systems, which at first glance showed a remarkable variation [9], [10]. Nevertheless, these results have to be put into perspective with regard to the options that alternative methods have to offer. Especially methods of subjective judgements score worse in comparison to objective measurement methods [16]. Even if the measurements in three-dimensional imaging are more variable than hoped for, there is usually no alternative method that provides better data [17]. The results obtained with the Visia^®^ camera here were based on a two-dimensional recording of skin surface characteristics, which might have contributed to the better precision in comparison with the above mentioned, objective capture systems. Differences of the data between 2% and 6% as obtained in this study overall indicate satisfactory precision of the available images. However, it remains to be discussed what size of differences are judged as good or satisfactory. 

Furthermore, once the precision of a capture system is established, future results of a clinical evaluation can be put into perspective. It is not a requirement that an objective camera system actually has a high degree of reproducibility, although this would be desirable, but that the magnitude of variability should be known in order to relativise later studies. Therefore, a clinical application should be preceded by a validation study [9], [12]. 

One question might be why the repetition of the captures in the same pose was not chosen to be more frequent. The answer lies in the relatively slow speed of the capture system and the time that is needed to reload the flashes for the repeated captures. The later restricted the use of this commercial system for research purposes. Since this system was built for commercial purposes, the user-friendliness for special applications, such as in this study, proved to be limited. There was also quite a steep learning curve in the application of the Visia^®^ camera. In the meantime, the company has introduced a second modified camera system for research purposes. Technical progress opens up the possibility of further investigations and clinical applications of this imaging method in the future, which has great potential. 

In order to conduct a power calculation, it was necessary to determine the presence of clinically relevant differences, which were considered to be those differences that would be visible to the human eye. For this purpose, an additional investigation with subjective assessment of each of the repeatedly captured images was conducted. Interestingly, differences between distinct versions of the native digital images captured by the Visia^®^ camera that were objectively detected by the camera were so small that they were not visible with the human eye in a subjective assessment of the study participants. Instead, the flash functionality of the Visia^®^ camera is the feature that enables the visualisation of the various skin features. These characteristics, such as skin spots, the presence of multiple wrinkles, or unevenness in the skin texture, are displayed and highlighted onscreen in multiple colours by the corresponding software application. Prior to this subjective investigation, the researcher had already had the impression that it would be very difficult to carry out a check on the data provided by the Visia^®^ camera with the human eye; this subjective assessment of the images provided further support for this view. One reason for this difficulty is that the Visia^®^ camera is a high-resolution digital camera system that uses several flashes to visualise aspects of the skin that would otherwise remain hidden in the native images; the relevant measurements in doing so are determined via the calculations made by the software system. In a validation study, there are generally several different important criteria of interest, including both accuracy and reproducibility of the instrument undergoing validation [9]. While the present study successfully investigated the reproducibility of results produced by the system under investigation, it was found that it was very difficult, if not impossible, to verify the accuracy of the information provided by the system via inspection and subjective assessment of the repeated native images. Therefore, the company producing the Visia^®^ camera is invited to validate the accuracy of the Visia^®^ camera system and publish the results in an independent publication venue. Such a validation check would entail checking the software calculations, and these checks would be the purview of the company’s software engineers and mathematicians. With such a check in place, future studies could continue this line of investigation to examine further questions in detail, such as whether there might be differences in results between the sexes or between skin types, or whether skin hydration levels would impact the results. Modern imaging technology offers many avenues for exciting research on various questions that could be a point of interest in future studies.

## Conclusion

The precision of the Visia^®^ camera system was found to be satisfactory in this study. 

The Visia^®^ camera helped to visualise skin features beyond what is visible to the human eye. 

Thus, the Visia^®^ camera system provides new objective information on skin surface characteristics beyond what can be acquired through purely subjective assessments. It can play a useful role in providing objective follow-up for various treatments and in making independent comparisons.

## Notes

### Acknowledgment

I acknowledge Dr. Wolfgang Reimers for his support in the statistical analysis of this study. 

I would like to thank my colleagues, the front desk staff and nurses in the clinic, who put up with me during the study. 

### Ethical statement

All procedures performed in the study were in accordance with the ethical standards of the institutional and national research committee and with the 1964 Helsinki declaration and its later amendments or comparable ethical standards.

### Competing interests

The author declares that she has no competing interests. This was an independent investigation of the Visia^®^ camera system.

## Figures and Tables

**Table 1:**
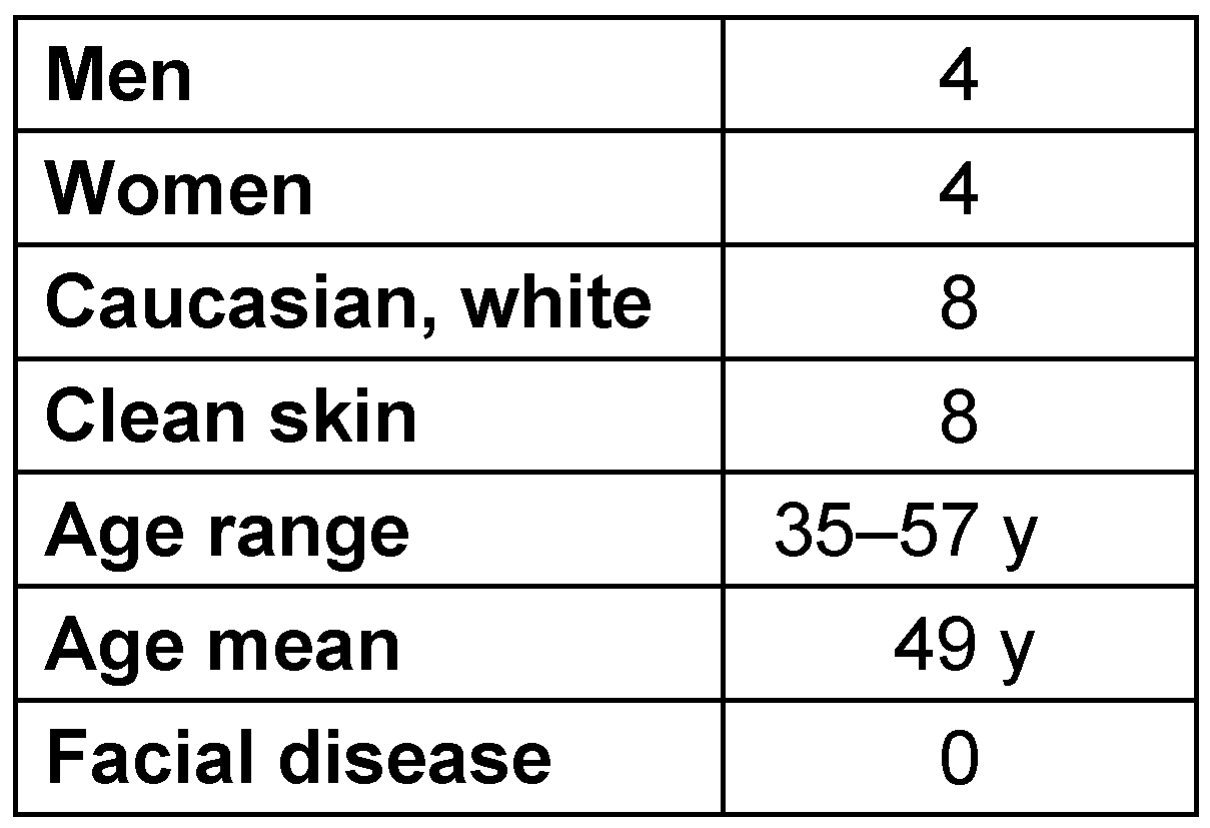
Demographic data of volunteers for facial capture

**Table 2:**
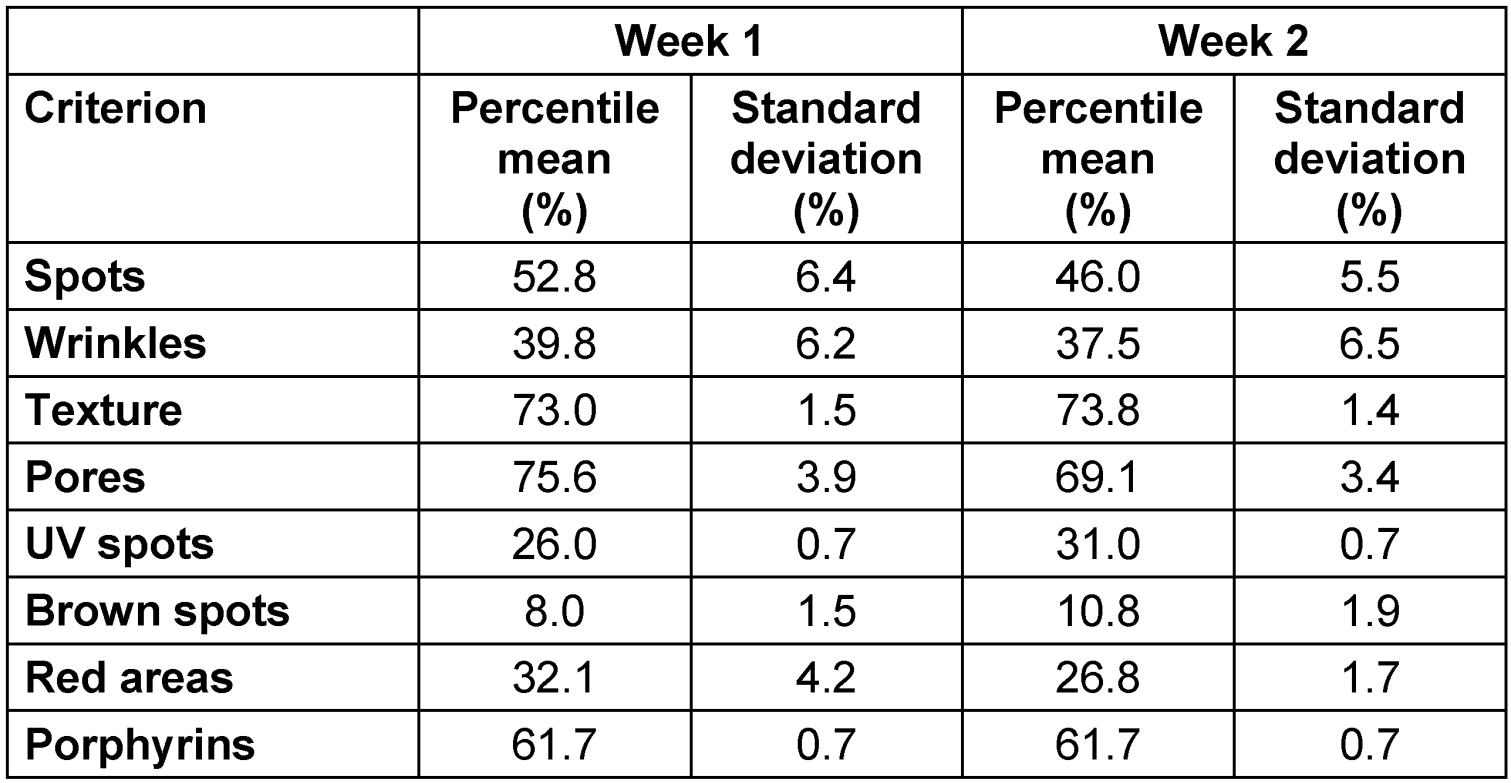
Percentile data as means and standard deviations for each criterion for week 1 and 2

## References

[1] Dietl M, Kompatscher P (2018). Basic Photographic Standards for Abdominal Contouring Procedures and Abdominoplasty/Lipectomy. Aesthetic Plast Surg.

[2] Hagan KF (2008). Clinical photography for the plastic surgery practice – the basics. Plast Surg Nurs.

[3] Rhee SC (2017). A Simple Method for International Standardization of Photographic Documentation for Aesthetic Plastic Surgery. Aesthetic Plast Surg.

[4] Bennett KG, Bonawitz SC, Vercler CJ (2019). Guidelines for the Ethical Publication of Facial Photographs and Review of the Literature. Cleft Palate Craniofac J.

[5] Kaliyadan F, Manoj J, Venkitakrishnan S, Dharmaratnam AD (2008). Basic digital photography in dermatology. Indian J Dermatol Venereol Leprol.

[6] Djian J, Lellouch AG, Botter C, Levy J, Burgun A, Hivelin M, Lantieri L (2019). Photographie clinique par smartphone en chirurgie plastique et protection des données personnelles: développement d’une plateforme sécurisée et application sur 979 patients. Ann Chir Plast Esthet.

[7] Nielson C, West C, Shimizu I (2015). Review of digital image security in Dermatology. Dermatol Online J.

[8] Henseler H (2011). Three-dimensional breast assessment by multiple stereophotogrammetry after breast reconstruction with latissimus dorsi flap [Ph.D. thesis].

[9] Henseler H, Khambay BS, Bowman A, Smith J, Paul Siebert J, Oehler S, Ju X, Ayoub A, Ray AK (2011). Investigation into accuracy and reproducibility of a 3D breast imaging system using multiple stereo cameras. J Plast Reconstr Aesthet Surg.

[10] Henseler H, Smith J, Bowman A, Khambay BS, Ju X, Ayoub A, Ray AK (2012). Investigation into variation and errors of a three-dimensional breast imaging system using multiple stereo cameras. J Plast Reconstr Aesthet Surg.

[11] Taheri A, Yentzer BA, Feldman SR (2013). Focusing and depth of field in photography: application in dermatology practice. Skin Res Technol.

[12] Henseler H, Kuznetsova A, Vogt P, Rosenhahn B (2014). Validation of the Kinect device as a new portable imaging system for three-dimensional breast assessment. J Plast Reconstr Aesthet Surg.

[13] Dissanayake B, Miyamoto K, Purwar A, Chye R, Matsubara A (2019). New image analysis tool for facial pore characterization and assessment. Skin Res Technol.

[14] Sapra S, Stewart JA, Mraud K, Schupp R (2015). A Canadian study of the use of poly-L-lactic acid dermal implant for the treatment of hill and valley acne scarring. Dermatol Surg.

[15] Kantikosum K, Chongpison Y, Chottawornsak N, Asawanonda P (2019). The efficacy of glycolic acid, salicylic acid, gluconolactone, and licochalcone A combined with 0.1% adapalene vs adapalene monotherapy in mild-to-moderate acne vulgaris: a double-blinded within-person comparative study. Clin Cosmet Investig Dermatol.

[16] Henseler H, Smith J, Bowman A, Khambay BS, Ju X, Ayoub A, Ray AK (2013). Subjective versus objective assessment of breast reconstruction. J Plast Reconstr Aesthet Surg.

[17] Henseler H, Hille-Betz U, Vogt PM (2015). Validierung subjektiver Schätzungen des weiblichen Brustvolumen und Vergleich zur objektiven Methode. Handchir Mikrochir Plast Chir.

